# Antioxidant, Enzyme Inhibitory, and Protective Effect of *Amelanchier lamarckii* Extract

**DOI:** 10.3390/plants13101347

**Published:** 2024-05-13

**Authors:** Adela Maria Dăescu, Mădălina Nistor, Alexandru Nicolescu, Roxana Pop, Andrea Bunea, Dumitrita Rugina, Adela Pintea

**Affiliations:** 1 Department of Chemistry and Biochemistry, University of Agricultural Sciences and Veterinary Medicine, Calea Mănăștur 3-5, 400372 Cluj-Napoca, Romania; adela.daescu@usamvcluj.ro (A.M.D.); nistor.madalina@usamvcluj.ro (M.N.); roxana.pop@usamvcluj.ro (R.P.); andrea.bunea@usamvcluj.ro (A.B.); 2 Laboratory of Chromatography, Institute of Advanced Horticulture Research of Transylvania, University of Agricultural Sciences and Veterinary Medicine, Calea Mănăștur 3-5, 400372 Cluj-Napoca, Romania; alexandru.nicolescu@usamvcluj.ro

**Keywords:** antioxidant, phenolics, *Amelanchier lamarckii*, bioaccessibility, enzyme inhibition, juneberries, retina

## Abstract

The present study aimed to investigate the chemical content of Romanian juneberries (*Amelanchier lamarckii*), their effect on antioxidant and enzyme inhibition activities, and their bioaccessibility after simulated in-vitro digestion. In *Amelanchier lamarckii* extract (AME), 16 polyphenolic compounds were identified by LC-ESI+-MS analysis. The most representative compounds found in the extract were cyanidin-galactoside, 3,4-dihydroxy-5-methoxybenzoic acid, feruloylquinic acid, and kaempferol, all belonging to the anthocyanins, phenolic acids, and flavonols subclasses. The polyphenols of AME exert quenching abilities of harmful reactive oxygen species, as the CUPRAC antioxidant assay value was 323.99 µmol Trolox/g fruit (FW), whereas the FRAP antioxidant value was 4.10 μmol Fe^2+^/g fruit (FW). Enzyme inhibition assays targeting tyrosinase (IC50 = 8.843 mg/mL), α-glucosidase (IC50 = 14.03 mg/mL), and acetylcholinesterase (IC50 = 49.55 mg/mL) were used for a screening of AME’s inhibitory potential against these key enzymes as a common approach for the discovery of potential antidiabetic, skin pigmentation, and neurodegenerative effects. The screening for the potential antidiabetic effects due to the α-glucosidase inhibition was performed in glucose-induced disease conditions in a human retinal pigmented epithelial cell experimental model, proving that AME could have protective potential. In conclusion, AME is a valuable source of phenolic compounds with promising antioxidant potential and metabolic disease-protective effects, warranting further investigation for its use in the nutraceutical and health industries.

## 1. Introduction

Plants of the *Amelanchier* genus, part of the *Rosaceae* family, are divided into 25 species that are native to Canada and North America. In the last two decades, species such as the Pacific or Saskatoon serviceberry (*Amelanchier alnifolia* (Nutt.) Nutt. ex M. Roem.), European juneberry or snowy Mespilus (*Amelanchier ovalis* Medik), Canadian serviceberry (*Amelanchier canadensis* L.), and juneberry (*Amelanchier lamarckii* F. G. Schroed.) have expanded to European countries such as Finland, Poland, the Czech Republic, and Romania [1]. The fruits of the genus *Amelanchier* have been used by indigenous peoples in traditional diets in the United States and Canada [2]. Lately, it has been confirmed that the fruits’ content is rich in bioactive compounds such as anthocyanins [3], flavonols, phenolic acids, flavones, minerals, and vitamins [4]. In this sense, it seems relevant to know the chemical composition of *Amelanchier* species’ fruits cultivated in Romania and to locally exploit their high nutritional value and health properties.

The antioxidant potential of *Amelanchier ovalis* against ABTS radicals was previously demonstrated and correlated to its high phenolic acid content [5]. A positive correlation between phenolic acids, flavan-3-ols, and antioxidant capacity was also observed by Lachowicz et al. (2017) [6]. The antioxidant potential of saskatoon berry (*Amelanchier alnifolia* Nutt.) cultivars (Thiessen and Smoky) was proved in an in vitro model, showing inhibition of peroxy-radical-induced intracellular oxidation in a concentration-dependent manner without affecting RAW264.7 macrophage viability [7]. The therapeutic potential of Saskatoon berry (*Amelanchier alnifolia*), containing anthocyanins, flavonols, ellagitannins, and phenolic acids, was determined in diet-induced metabolic syndrome. After the administration of Saskatoon berry powder for 16 weeks in male Wistar rats fed a high-carbohydrate or high-fat diet, a decrease in total plasma cholesterol and infiltration of inflammatory cells in the heart, as well as the normalization of body weight and adiposity and an increase in glucose tolerance, were observed [8]. Additionally, a reduction in postprandial blood glucose was registered in a mouse model of diet-induced obesity and hyperglycemia after the introduction of an extract of *Amelanchier ovalis* in their diet [9]. Recently, in another experiment performed on an animal model of high-fat, high-sucrose mice, a decrease in vascular inflammation, hyperlipidemia, and hyperglycemia was reported following the administration of a powder obtained from Saskatoon berry fruits for 15 weeks [10]. Taken together, these studies suggest that *Amelanchier* sp. has great anti-diabetic potential that should be further investigated.

The determination of the capacity of natural compounds to inhibit the activity of key enzymes such as α-glucosidase (EC 3.2.1.20), acetylcholinesterase (EC 3.1.1.7), and tyrosinase (EC 1.14.18.1) is currently used as a tool to evaluate the potential therapeutic effects of plant extracts. Inhibition of acetylcholinesterase, the enzyme that hydrolyzes acetylcholine to choline and acetate, could preserve levels of acetylcholine and the cholinergic function, thus improving the status of patients with Alzheimer’s disease [11]. *Amelanchier ovalis* subsp. ovalis has been reported to exert significant anticholinesterase properties [12]. Zengin et al. (2018) observed that *Amelanchier parviflora* var. Dentata’s ethanolic and methanolic extracts exert anticholinesterase inhibitory activities [13].

α-glucosidase is a membrane-bound intestinal enzyme that hydrolyzes saccharides to liberate glucose and, therefore, a key enzyme for glycaemic control [13]. *Amelanchier parviflora* var. Dentata’s ethanolic and aqueous extracts actively inhibited α-glucosidase, but no activity was observed for the methanolic extract [13]. An inhibition effect on α-glucosidase was exerted by seven Saskatoon *Amelanchier alnifolia* berry fruits and their fractions [14]. The Saskatoon berry extract inhibits α-glucosidase and affects glucose uptake through the insulin-like effect [15].

Tyrosinase is a copper-containing oxidase that plays various roles in living organisms, including melanogenesis and enzymatic browning [16]. Similarly to AChE inhibition, methanolic *Amelanchier parviflora* var. Dentata extract exerted the highest inhibitory capacity against tyrosinase, compared to its ethanolic and aqueous extracts [13].

The digestibility and biological properties of antioxidants are usually determined by the use of various in vitro digestion models. The bioaccessibility and bioavailability of phenolic compounds are influenced by many factors, starting with the chemical features, the type of food matrix, the food processing, the interaction with other food constituents, and the host-related factors [17]. Although in vivo studies remain the gold standard for the evaluation of bioavailability, in vitro digestion protocols can give valuable insights regarding the bioaccessibility of natural compounds. In the present study, the INFOGEST standardized in vitro digestion protocol was used [18]. In a previous study, a fortified rye bread supplemented with 3% of the functional Saskatoon berry fruit powder was subjected to simulated in vitro digestion and showed that anthocyanins were highly bioaccessible compounds, whereas the least bioaccessible turned out to be flavan-3-ols [19].

Considering all the facts discussed above, the present study aimed to determine the chemical composition of *Amelanchier lamarckii* berries cultivated in Romania, to provide original and valuable information for the nutraceutical and health industries regarding the antioxidant activity and enzymatic inhibitory activity of the fruit extract, along with the determination of the bioaccessibility of major bioactive phenolic compounds.

## 2. Results and Discussion

### 2.1. Identified Polyphenolic Compounds

The identification of the polyphenolic compounds was carried out by LC-ESI^+^-MS analysis, based on maximum absorption, elution order, MS signals (molecular peaks and characteristic fragments), and the literature’s existing data. A total of 16 polyphenolic compounds were identified in the fruits of *Amelanchier lamarckii* (AME) (see Table 1). Of these compounds, the dominant ones were hydroxybenzoic and hydroxycinnamic acids in concentrations of 476.77 μg/g fruit (FW) and 436.22 μg/g fruit (FW). In the extract were also found anthocyanins (347.24 μg/g fruit (FW)) and flavonols (449.29 μg/g fruit (FW)), such as quercetin, quercetin dirhamnoside, quercetin rhamnoside, kaempferol, kaempferol glucoside, and kaempferol rhamnoside. The total calculated polyphenol content in AME was 1800.32 ± 6.56 μg/g fruit (FW). In *Amelanchier lamarckii* cultivated in Slovenia, the identified phenolic composition included hydroxycinnamic acid and hydroxybenzoic acid derivatives, anthocyanins, flavones, flavanols, and flavonol derivatives [1]. The phenolic amounts in this study are different (about 90 mg phenolic compounds/kg berries), being expressed on dry weight, and the moisture content of the berries is not given. Anthocyanins ranged between 258.7 and 517.9 mg/100 g of fresh weight in different Saskatoon berries (*Amelanchier alnifolia* Nutt.), which accounted for 63% of the small molecular weight phenolics [20]. The ripe fruits of *Amelanchier canadensis* (L.) Medik. contained phenolic compounds of catechins (343.46 ± 29.46 mg/100 g FW), anthocyanins (220.66 ± 17.43 mg/100 g FW), and cinnamic acids (209.29 mg/100 g FW) [3]. Previous studies have underlined that variations in the composition of phytochemicals in plants are influenced by physiological, genetic, and environmental factors [21].

### 2.2. Antioxidant Activity

The quenching capacity of the identified polyphenols in the extract was evaluated by performing two antioxidant activity assays. It is recommended that at least one combination of two assays based on electron transfer mechanisms be used to provide accurate data regarding the antioxidant activity of the sample. Here, two antioxidant methods, CUPRAC and FRAP, both based on electron transfer mechanisms, have been used. The AME quenching potential determined by the FRAP method was 4.10 μmol Fe^2+^/g (FW). The antioxidant potential of AME analyzed by the CUPRAC assay was found to be 323.99 µmol Trolox/g (FW).

*Amelanchier lamarckii* var. Ballerina berries collected from two different production years (2012 and 2013) generated similar values, according to FRAP analysis: 0.88 ± 0.01 mmol Fe^2+^/kg (2012) and 0.85 ± 0.0 mmol Fe^2+^/kg (2013) for the DMSO extract; 0.96 ± 0.0 mmol Fe^2+^/kg (2012) and 1.02 ± 0.01 mmol Fe^2+^/kg (2013) for the ethanolic (50%) extract; and 1.0 ± 0.0 mmol Fe^2+^/kg (2012) and 0.91 ± 0.0 mmol Fe^2+^/kg (2013) for the aqueous extract [22]. The antioxidant activity of the Polish clone type S of *Amelanchier alnifolia* Nutt. was evaluated by the FRAP assay as being 34.49 and 25.34 mmol Trolox/100 g DM [6]. The difference between these results and our data may come from the water content present in the fresh fruit and dry weight samples used. *Amelanchier parviflora* var. Dentata species investigated for antioxidant potential had CUPRAC values of 506.18 ± 3.85 mg Trolox/g extract and FRAP values of 274.01 ± 9.36 mg Trolox/g extract [13].

### 2.3. Key Enzyme Inhibition

**Figure 1:**
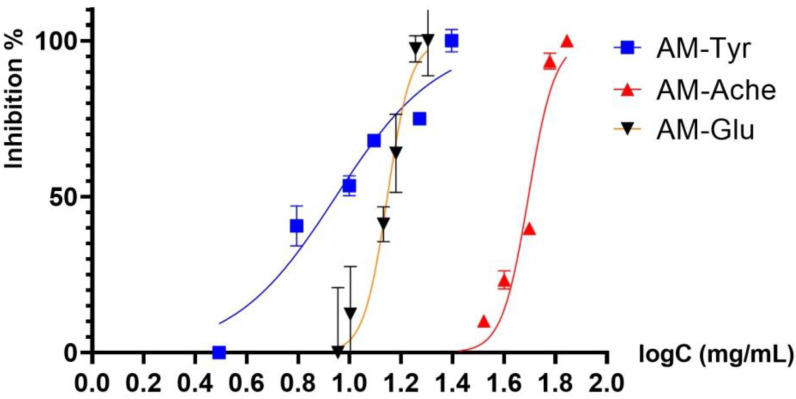
Inhibition of tyrosinase (tyr), α-glucosidase (glu), and acetylcholinesterase (ache) after 15 min of incubation with *Amelanchier lamarckii* extract (AME). The plots represent the normalized logarithmic concentrations versus percentages of inhibition.

The AME inhibitory potential for targeting the key enzymes α-glucosidase, tyrosinase, and acetylcholinesterase (Figure 1) was used for the discovery of molecules with potential anti-skin pigmentation, antidiabetic, and anti-neurodegenerative effects.

Tyrosinase is considered a critical therapeutic target for treating skin disorders caused by the overproduction of melanin [23]. Kojic acid is a well-known tyrosinase inhibitor, a depigmenting agent commonly used as a positive control for screening extracts that effectively inhibit melanin synthesis. Overstimulation of the biosynthesis of melanin leads to pigmentation disorders, like melasma or even skin cancer [24]. As shown in Table 2, the AME inhibitory activity on tyrosinase exerts an IC50 of 8.84 mg/mL, meaning that it has a lower anti-tyrosinase potential than the kojic acid (IC50 of 0.02 mg/mL) standard. The inhibition activity of tyrosinase by polyphenols like quercetin, isorhamnetin, and gallic acid was previously reported [25]. The extract obtained from *Amelanchier parviflora* var. Dentata was proved to exert anti-tyrosinase (145.54 mg kojic acid equivalents/g extract) and anti-acetylcholinesterase (3.63 mg galantamine equivalents/g extract) activity [13].

α-glucosidase has a key role in carbohydrate metabolism. The rapid postprandial increase in blood glucose levels can be delayed by inhibiting α-glucosidase activity, which is considered helpful in preventing type 2 diabetes [26]. Here, AME proved to inhibit α-glucosidase with an IC50 of 14.03 mg extract/mL (Table 2), but had a lower inhibitory potential than the positive control, a synthetic inhibitor widely used as an anti-diabetic drug, known as acarbose (0.49 mg extract/mL solution). In a recent study, the chemical structure of nine phenolic acids (caffeic acid, ferulic acid, gallic acid, protocatechuic acid, p-coumaric acid, syringic acid, sinapic acid, vanillic acid, and chlorogenic acid) was analyzed to determine if it influences their interaction with substrates or enzymes (α-amylase and α-glucosidase). Results indicated that the presence of methoxyl groups on benzoic acid derivatives affected their capacity to interact with the substrates for both α-amylase and α-glucosidase [27]. Moreover, via molecular docking analysis, it was observed that the removal of hydroxyl groups of polyphenols may decrease the inhibition effect [27,28].

The inhibitory action of serviceberry leaf extracts against α-glucosidase activity and its effects on intestinal absorption of carbohydrates in an animal model of obesity and hyperglycemia were previously evaluated. Studies proved that the serviceberry leaf subfraction inhibited intestinal α-glucosidase activity and delayed the absorption of carbohydrates, resulting in a significant lowering of postprandial blood glucose concentrations, similar to the antidiabetic drug acarbose [9].

Acetylcholinesterase has a key role in catalyzing the hydrolysis reaction of the acetylcholine neurotransmitter after the nerve impulse passes the synapse, producing choline and acetate. Acetylcholinesterase has a significant role in controlling and treating neurodegenerative diseases [29]. AME was proved to have anti-acetylcholinesterase activity with an IC50 measured at 49.55 mg extract/mL solution (Table 2), but it is lower than that of the galantamine positive control. In the literature, there are data regarding the polyphenols’ impact on acetylcholinesterase activity. For example, extracts such as *Centarium umbellatum* showed a high acetylcholinesterase inhibitory effect of about 94.24% (for the highest tested concentration, 3 mg/mL), whereas the extract obtained from Pulmonaria officinalis showed slightly lower acetylcholinesterase inhibitory effects (87.7%), thus being considered potential therapeutic sources with anti-acetylcholinesterase activity [30].

### 2.4. Simulated In Vitro Digestion Model

The bioaccessibility of polyphenolics was investigated by replicating physiological conditions through simulated in vitro digestion. The total concentration of polyphenols in AME decreased from 1800.32 ± 7.27 to 268.01 ± 1.73 μg/g FW after the gastric phase and subsequently to 182.62 ± 0.82 μg/g FW after the intestinal phase (Table 3). The total bioaccessibility of polyphenols calculated was about 10.14%, which indicates that only a small fraction of the polyphenols present in the extract was accessible for absorption. The reduced bioavailability of polyphenols can be explained by the chemical reactions and transformations under different pH conditions, and the slightly alkaline environment of the small intestine [31,32]. Among different types of polyphenols, isoflavones are the most readily absorbed, followed by phenolic acids, flavanols, flavanones, flavonols, anthocyanins, and proanthocyanidins [33]. Flavonoids, which are hydrophilic compounds, generally have low bioavailability due to their poor absorption in the gut [34]. These facts are consistent with our results, where the flavone subclass had the highest concentration, namely 32.90% of the initial concentration after the gastric phase, but none could be identified after the intestinal phase. The other compounds had similar bioaccessibility values in the gastric phase, with anthocyanins measuring the lowest concentration of all the subclasses (4.05%). Regarding the intestinal phase, hydroxycinnamic acid showed the highest concentration identified (17.09%), followed by flavonols (15.41%) and hydroxybenzoic acid (8.14%). However, no traces of anthocyanins or flavones could be observed after the intestinal phase, possibly due to their instability during the digestion process, especially at alkaline pH [35]. The very low bioaccessibility/bioavailability of anthocyanins is a known issue, with values below 0.1% of the intake being reported [36]. Interestingly, Lakowicz and coworkers (2020) found superior bioaccessibility for anthocyanins from rye bread fortified with free and microencapsulated powders (up to 6.4%) from *Amelanchier alnifolia* Nutt. compared to flavan-3-ol, which was explained by the effect of carriers (maltodextrin and inulin) [19].

### 2.5. Extract Effects on RPE Cells Exposed to High-Glucose Conditions

Due to the potential use of the anti-α-glucosidase effect of AME as an alternative strategy in diabetes, we made use of the disease simulation conditions in human retinal epithelial cells (RPE cells) to evaluate the antiproliferative effects of AME. It is known that prolonged exposure of retinal pigmented epithelial cells to hyperglycemia contributes to the development of diabetic retinopathy [37].

**Figure 2:**
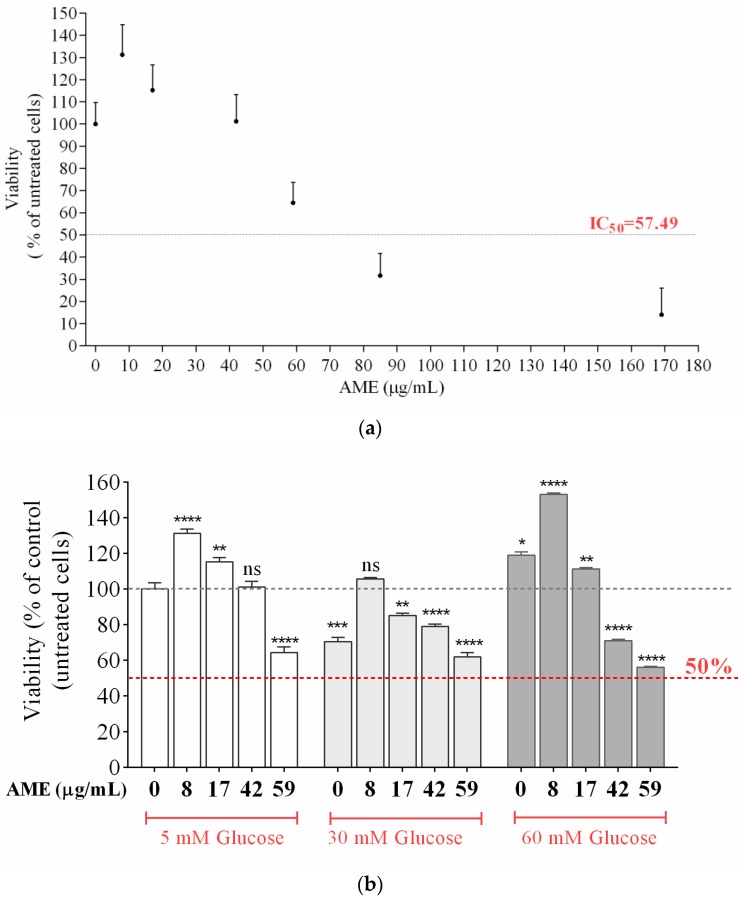
Influence of AME (µg/mL) on human pigmented epithelial D407 cells’ proliferation in basal glucose (5 mM) conditions (**a**). The effect of AME (µg/mL) on D407 cells in different glucose-concentration environments (5, 30, and 60 mM). Results are presented as the mean ± SD of 5 measurements, one-way ANOVA, and multiple comparison Dunnett’s test (**b**). The data were compared using the one-way ANOVA test. **** means *p* < 0.0001, and *** means *p* < 0.001, ** means *p* < 0.01, * means *p* < 0.01, indicating highly significant differences between the sets of data.

A dose-response curve and the half-maximal inhibitory concentration (IC50) after 24 h exposure to AME (0–170 µg/mL) is represented in Figure 2a. The determined IC50 after 24 h exposure to AME is 57.49 µg/mL, and the dose-response curve proves a decrease in cell viability with increasing AME concentration.

In the cell culture media containing high-glucose conditions of 30 mM and 60 mM glucose, AME doses ranging from 0 to 17 µg/mL prevented the loss of cell viability, suggesting a protective effect of the extract (Figure 2b). The anti-diabetic action of the *Amelanchier* genus has been previously observed in a study on obese, hyperglycemic C57Bl6 mice [9].

## 3. Materials and Methods

### 3.1. Reagents

Chromatographic analysis: methanol and acetonitrile were bought from Chempur, and formic acid from Merck KGaA (Darmstadt, Germany).

Antioxidant assays: 2,4,6-Tris(2-pyridyl)-triazine (TPTZ), 6-hydroxy-2,5,7,8-tetramethylchroman-2-carboxylic acid (Trolox) (98% purity), 2,9-dimethyl-1,10-phenanthroline (Neocuproine), and Copper (II) chloride (CuCl_2_) were bought from Sigma-Aldrich (St. Louis, MO, USA), purchased from Sigma Chemical Co. (St. Louis, MO, USA).

Enzyme inhibition assay: α-glucosidase, tyrosinase, acetylcholinesterase, acarbose, kojic acid, Tris-HCl buffer, galantamine, DTNB (Ellman’s Reagent) (5,5-dithio-bis-(2-nitrobenzoic acid), ATCI (acetyl-thiocholine iodide), L-DOPA (L-3,4-dihydroxyphenylalanine), and PNPG (4-Nitrophenyl-β-D-glucopyranoside) were obtained from Sigma-Aldrich (St. Louis, MO, USA).

In vitro digestion: α-amylase from human saliva (P6887), pepsin from porcine gastric mucosa (P6887), pancreatin from porcine pancreas (P7545), and bovine bile extract (B3883) were bought from Sigma-Aldrich (Steinheim, Germany).

Other materials: acetate buffer (pH 3.6), phosphate buffer (PBS), NaOH, FeCl_3_, and Tris HCl (Tris hydrochloride) were purchased from local suppliers.

For the cell culture experiments, the following materials were purchased: human retinal pigment epithelium D407 (a generous offer from Prof. Em Horst Diehl of Universität Bremen); low-glucose DMEM (Biowest, Nuaillé, France); PBS 1x (Gibco, Waltham, MA, USA); Trypsin 0.05%/EDTA (PAN-Biotech GmbH, Aidenbach, Germany); L-Glutamine and Antibiotic Mix (Gibco, Waltham, MA, USA); Fetal Bovine Serum (Gibco, Waltham, MA, USA); glucose solution (Gibco, Waltham, MA, USA), and WST-1 assay (Roche Applied Science, Basel, Switzerland). The plastic ware was purchased from Eppendorf (Hamburg, Germany) and VWR (Radnor, PA, USA).

### 3.2. Obtaining the Polyphenolic Extract

The fruits of *Amelanchier lamarckii* shrubs were collected at full maturity from Cluj-Napoca, Romania and stored at −80 °C. Then, 5 g of fresh fruits was finely minced using an Ultraturax (model Miccra D-9 KT; Digitronic GmbH, Bergheim, Germany) in acidified methanol (0.3% with HCl). The extract was evaporated at 40 °C to dryness using a rotary evaporator (Rotavapor^®^ model R-124; Buchi, Basel, Switzerland). The *Amelanchier lamarckii* concentrated extract (AME) was finally dissolved in acidified methanol, filtered through 0.2 μm, and then stored at −20 °C until analysis. The voucher specimen (Number 30411) was deposited at the Herbarium of the University of Agricultural Sciences and Veterinary Medicine, Cluj-Napoca.

### 3.3. LC-ESI^+^-MS Analysis

Samples were analyzed by an Agilent Technologies 1200 HPLC system (Chelmsford, MA, USA) equipped with a G1311A quaternary pump, a G1322A degasser, a G1329A autosampler, and a G1315D photo-diode array detector. In-line MS data were recorded by directing the LC flow to a Quadrupole 6110 mass spectrometer (Agilent Technologies, Chelmsford, MA, USA) equipped with an ESI probe. Polyphenols were detected using the positive ionization mode, with several fragments operating in the 50–100 V range. The column was a Kinetex XB-C18 from Phenomenex (5 µm; 150 × 4.6 mm i.d.). Water (0.1% acetic acid) (solvent A) and acetonitrile (0.1% acetic acid) (solvent B) were used as the mobile phases. The following multistep linear gradient was used: 5% B for 2 min, 5–90% B for 20 min, 90% B for 4 min, and 5% B for 6 min. The flow rate was 0.5 mL/min, and the oven temperature was 25 °C. Chromatograms were recorded at λ = 280 nm, 340 nm, and 520 nm. Quantification of major polyphenols was performed by external calibration with commercial standards of gallic acid (5–100 µg/mL), chlorogenic acid (5–50 µg/mL), catechin (5–200 µg/mL), rutin (10–100 µg/mL), and cyanidin (5–100 µg/mL), and was repeated three independent times.

### 3.4. Antioxidant Activity Assays

The reduction of ferric 2,4,6-tris(2-pyridyl)-1,3,5-triazine [Fe (III)-TPTZ] by AME to form a blue ferrous complex at low pH was determined by the FRAP test according to the protocol previously published [38]. Briefly, the working solution was prepared by mixing 10 mM/L TPTZ with 20 mM ferric chloride in acetate buffer (pH 3.6). Then, 20 μL of the AME was mixed with 180 μL of the working solution. The absorbance of the sample was read at 593 nm after 30 min of incubation at 37 °C. The results were calculated and expressed as μmol Fe^2+^/g fruit (FW), based on the calibration curve (y = 1.304x − 0.0085, R2 = 0.9992).

The cupric ion-reducing antioxidant capacity (CUPRAC) of the extract was determined according to a previously described method [39]. The absorbance of the sample was read at 450 nm against the blank with a JASCO V-630 spectrophotometer (International Co., Ltd., Tokyo, Japan). The antioxidant activity was calculated in µmol Trolox equivalents/g fruit (FW), based on the calibration curve (y = 0.0492x − 0.0497, R2 = 0.9866), and was repeated three times.

### 3.5. Enzyme Inhibitory Potential

The inhibitory potential of AME was evaluated against the following enzymes: α-glucosidase, tyrosinase, and acetylcholinesterase. Readings were carried out with 96-well plates and a SPECTROstar Nano Multi-Detection microplate reader (BMG Labtech, Ortenberg, Germany), being repeated three independent times.

#### 3.5.1. Tyrosinase

Tyrosinase inhibitory activity was analyzed following the previously published method [40]. Four wells for each sample were prepared as follows: (A) Control: 120 μL PBS and 40 μL tyrosinase; (B) Control blank: 160 μL PBS; (C) Sample: 40 μL AME, 80 μL PBS (5 mM, pH 6.8), and 40 μL tyrosinase; and (D) Sample blank: 40 μL AME and 120 μL PBS. After incubating the plate at 25 °C for 10 min, each well received 40 μL of the substrate L-DOPA (10 mM) in PBS solution to a final volume of 200 μL. The plate was then immediately incubated at 25 °C for 20 min. Using kojic acid as a positive control, the absorbance of each sample was measured at 475 nm. The AME sample used here was evaporated, and 24 mg (for the tyrosinase assay) was redissolved in 1 mL of water containing 5% DMSO to obtain a stock solution, from which the other concentrations were obtained (concentrations 3, 6, 9, 12, 18, and 24 mg/mL).

#### 3.5.2. α-Glucosidase

The α-glucosidase inhibition test was performed based on a slightly modified protocol previously published [41]. In a nutshell, four samples were prepared: (A) Control: 100 μL PBS and 50 μL enzyme in PBS; (B) Control blank: 150 μL PBS; (C) Sample: 50 μL AME with 50 μL PBS (0.1 M pH 6.8) and 50 μL enzyme in PBS; and (D) Sample blank: 50 μL AME and 100 μL PBS. After 15 min of incubation at 37 °C, each sample received 50 μL of substrate (PNPG, 10 mM in PBS) to a final volume of 200 μL. The absorbance of the final reaction mixture was measured at a wavelength of 405 nm. Acarbose, the synthetic oral hypoglycemic agent, was used as a positive control. The AME sample used here was evaporated, and 24 mg (for the glucosidase assay) was redissolved in 1 mL of water containing 5% DMSO to obtain a stock solution, from which the other concentrations were obtained (concentrations 9, 10, 13, 15, 18, and 20 mg/mL).

#### 3.5.3. Acetylcholinesterase

The protocol for the acetylcholinesterase inhibition test was modified based on Ellman’s protocol [42,43]. Four samples were prepared, as follows: (A) Control: 75 μL Tris-HCl buffer, 125 μL DTNB, and 25 μL of enzyme solution; (B) Control blank: 100 μL Tris-HCl buffer and 125 μL DTNB. (C) Sample: 25 μL AME, 50 μL Tris-HCl buffer (50 mM, pH 8.0), 125 μL of DTNB substrate (0.9 mM) in Tris-HCl buffer, and 25 μL of enzyme solution (with an activity of 0.078 U/mL, in buffer); and (D) Sample blank: 25 μL diluted extract, 75 μL Tris-HCl buffer, and 125 μL DTNB. After 15 min of dark incubation at 25 °C, 25 μL of an ATCI solution (4.5 mM) was added, and the mixture was then re-incubated for 10 min. Galantamine, the best inhibitor against cholinesterase, was used as a positive control, and the absorbance was measured at 405 nm. The AME sample used here was evaporated, and 24 mg (for the acetylcholinesterase assay) was redissolved in 1 mL of water containing 5% DMSO to obtain a stock solution, from which the other concentrations were obtained (concentrations 26, 33, 39, 49, 60, and 69 mg/mL).

To calculate the inhibition percentage of each enzyme, the following formula has been used:

$$\%\;I=\frac{{Abs}_{\left(A-B\right)}-{Abs}_{\left(C-D\right)}}{{Abs}_{\left(A-B\right)}}\times 100$$

where (*A*) Control; (*B*) Control blank; (*C*) Sample; and (*D*) Sample blank.

### 3.6. Simulated In Vitro Digestion

In order to simulate in vitro gastrointestinal digestion, the method described by Minekus et al. (2014) was used [18]. The fruit samples were subjected to the in vitro static digestion methodology (INFOGEST), which consists of three phases: oral, gastric, and small intestinal.

The oral phase: 2.8 g of finely ground fruit samples was mixed with 1.9 mL of simulated salivary fluid (SSF), 150 μL of CaCl_2_, and 0.5 mL of α-amylase in SSF (75 units/mL in the final volume). To the mixture that resulted, water was added to the final volume of 6 mL. Next, the mixture was incubated for 2 min at 37 °C in a shaking water bath (Memmert GmbH Co. KG, Schwabach, Germany).

The gastric phase: 3.8 mL of simulated gastric fluid (SGF), 30 μL of CaCl_2_ (0.03 M), and 1 mL of porcine pepsin in simulated gastric fluid (SGF) (2000 U/mL in final volume) were added to the oral bolus. The pH was adjusted to 3.0 using concentrated formic acid, and 1 mL of water was added to make the final volume of 12 mL. The final mixture was incubated at 37 °C for 2 h in a water bath set at 150 orbital shakes per minute.

The small intestinal phase: To mimic the conditions of the duodenum, the gastric chyme was mixed with 5.6 mL of simulated intestinal fluid (SIF), 120 μL of CaCl_2_ (0.03 M), 2 mL of pancreatin in SIF (2000 U/in final volume), and 2 mL of bile salts in SIF (10 mM in final volume). The final digesting mixture was adjusted to a pH of 7.0 with NaOH (1 M), and then water was added to obtain the desired final volume of 24 mL. The samples were incubated in a shaking water bath for 2 h at 37 °C.

Finally, the total digesta was centrifuged (Eppendorf 5810 R, Hamburg, Germany) at 10,000 rpm (12,298× *g*) for 90 min at 4 °C. The supernatant of each sample was collected and adjusted to a pH of 3 before HPLC analysis. The bioaccessibility was calculated using the following formula and represents the percentage of the total polyphenol concentration in the analyzed samples (digesta) to the total initial content of polyphenols extracted from raw materials:

$$\mathrm{Bioaccessibility}\;(\%)=\frac{Total\;polyphenols\;content\;in\;digesta}{Total\;initial\;polyphenols\;in\;fruit\;samples\;subjected\;to\;digestion}\times 100$$

The same formula was used to calculate the bioaccessibility of different polyphenol classes. The experiment was repeated three times.

### 3.7. In Vitro Studies on Retinal Pigmented Epithelial Cells

Cell culture: The human retinal pigment epithelial cell line D407 was maintained at 37 °C, 5% CO_2_, and 95% relative humidity.

Cytotoxicity assay: Cell viability was determined by using the WST-1 reagent from Roche (Basel, Switzerland). After seeding, 8 × 103 cells/well were treated with AME (0 to 170 µg/mL concentrations) in cell culture medium for 24 h. For the high-glucose stress-induced test, the treatment for D407 cells was glucose (5, 30, and 60 mM in cell culture medium) with or without AME (0, 8, 17, 42, or 59 µg/mL) for 24 h. Afterward, WST-1 reagent (15 µL) was applied to the cells and incubated for 30 min. The absorbances were recorded using a plate reader (Biotek Synergy HT, Winooski, VT, USA) at wavelengths of 450 and 600 nm (background). The viability experiment was done in triplicate, each time including 5 repetitions.

## 4. Conclusions

Of the polyphenols of Romanian juneberries *Amelanchier lamarckii*, phenolic acids, anthocyanins, flavonols, and flavones prove to have a great contribution to the antioxidant activity potential and enzymatic inhibitory potential on tyrosinase, α-glucosidase, and acetylcholinesterase. The total concentration of polyphenols in AME was decreased six times after the gastric phase compared to the initial AME and ten times after the intestinal phase. The total bioaccessibility of the polyphenols indicates that only a small fraction of them is accessible for absorption, maybe due to the chemical transformations under different pH conditions and the slightly alkaline environment of the small intestine. Flavonols and hydroxycinnamic acids had the highest bioaccessibility, while anthocyanins and flavones could not be detected in the final digesta.

In a simulated diabetic retinopathy model, polyphenols of AME exerted a protective effect in high-glucose conditions of 30 mM glucose. However, in extreme glucose conditions (60 mM glucose), the protective effect of AME on the viability of D407 cells was reduced.

Taken together, all these data suggest that *Amelanchier lamarckii* might be a valuable source of polyphenolic compounds with antioxidant potential and metabolic disease-protective effects worth being further investigated and exploited in the food and nutraceutical industries.

## Figures and Tables

**Table 1:** Polyphenolic compounds identified and quantified in *Amelanchier lamarckii* extract (AME) by LC-ESI^+^-MS analysis.

Peak	Rt (min)	UV λ_max_ (nm)	[M + H]^+^ (*m*/*z*)	Compound	Subclass	Concentration (μg/g) Fruit (FW)
1	7.37	280	185	3,4-Dihydroxy-5-methoxybenzoic acid	Hydroxybenzoic acid	281.32 ± 0.53
2	10.62	280, 515	449, 287	Cyanidin-galactoside	Anthocyanin	308.55 ± 0.79
3	12.44	332	355	Chloro genic acid	Hydroxycinnamic acid	93.83 ± 0.37
4	13.18	270, 320	595, 287	Luteolin-rutinoside	Flavone	19.18 ± 0.41
5	13.47	280, 520	287	Cyanidine	Anthocyanin	38.69 ± 2.69
6	13.48	280	305, 141	4-Hydroxybenzoic acid-glucoside	Hydroxybenzoic acid	195.45 ± 0.53
7	15.07	330	369	Feruloylquinic acid	Hydroxycinnamic acid	164.75 ± 0.78
8	15.15	330	369	Feruloylquinic acid	Hydroxycinnamic acid	145.20 ± 0.48
9	15.89	250, 360	596, 303	Quercetin-dirhamnoside	Flavonol	87.49 ± 0.50
10	16.44	250, 360	449, 303	Quercetin-rhamnoside	Flavonol	6.69 ± 0.50
11	18.59	260, 350	449, 287	Kaempferol-glucoside	Flavonol	61.46 ± 1.00
12	19.71	322	517, 163	Dicaffeoylquinic acid	Hydroxycinnamic acid	32.44 ± 0.66
13	20.41	260, 350	433, 287	Kaempferol-rhamnoside	Flavonol	119.24 ± 0.59
14	21.13	270, 320	287	Luteoline	Flavone	71.63 ± 4.55
15	21.32	250, 360	303	Quercetin	Flavonol	14.42 ± 0.20
16	23.19	260, 350	287	Kaempferol	Flavonol	159.98 ± 2.17
				Total polyphenols		1800.32 ± 6.56

**Table 2:** Half-maximum inhibitory concentrations of *Amelanchier lamarckii* extract (AME) against tyrosinase, α-glucosidase, and acetylcholinesterase and their corresponding positive controls, acarbose, kojic acid, and galantamine.

Sample/Standard	Tyrosinase IC50 (mg/mL)	α-Glucosidase IC50 (mg/mL)	Acetylcholinesterase IC50 (mg/mL)
AME	8.84	14.03	49.55
Acarbose	-	0.49	-
Kojic acid	0.02	-	-
Galantamine	-	-	0.04

**Table 3:** Bioaccessibility of phenolic compounds from *Amelanchier lamarckii* berry extract (AME) after simulated gastro-intestinal digestion. The italics are used to underline that the values represent bioaccessibility, while the upper values (not in italics) are the concentrations based on which the bioaccessibility is calculated.

Subclass of Polyphenolic Compounds	Concentration (μg/g Fruit (FW)) *Bioaccessibility* (%)		
	AME	Gastric Phase	Intestinal Phase
Hydroxybenzoic acids	476.77 ± 0.45	94.53 ± 0.50 *19.83%*	38.83 ± 0.43 *8.14%*
Anthocyanins	347.24 ± 2.22	14.07 ± 0.56 *4.05%*	nd
Hydroxycinnamic acids	436.22 ± 0.65	81.25 ± 0.41 *18.63%*	74.56 ± 0.53 *17.09%*
Flavones	90.81 ± 2.81	29.88 ± 0.38 *32.90%*	nd
Flavonols	449.29 ± 2.04	48.27 ± 0.70 *10.74%*	69.23 ± 0.92 *15.41%*
Total	1800.32 ± 7.27	268.01 ± 1.73 *14.89%*	182.62 ± 0.82 *10.14%*
Bioaccessibility	10.14%		

## Data Availability

The original contributions presented in the study are included in the article, further inquiries can be directed to the corresponding authors.
